# Intracellular Signaling in Key Pathways Is Induced by Treatment with Ultrasound and Microbubbles in a Leukemia Cell Line, but Not in Healthy Peripheral Blood Mononuclear Cells

**DOI:** 10.3390/pharmaceutics11070319

**Published:** 2019-07-06

**Authors:** Ragnhild Haugse, Anika Langer, Stein-Erik Gullaksen, Silje Maria Sundøy, Bjørn Tore Gjertsen, Spiros Kotopoulis, Emmet McCormack

**Affiliations:** 1 Department of Clinical Science, The University of Bergen, Jonas Lies vei 65, 5021 Bergen, Norway; 2 Department of Quality and Development, Hospital Pharmacies Enterprise in Western Norway, Møllendalsbakken 9, 5021 Bergen, Norway; 3 Centre for Cancer Biomarkers CCBIO, Department of Clinical Science, The University of Bergen, Jonas Lies vei 65, Bergen 5021, Norway; 4 Department of Internal Medicine, Hematology Section, Haukeland University Hospital, Jonas Lies vei 65, 5021 Bergen, Norway; 5 Phoenix Solutions AS, Ullernchausseen 64, 0379 Oslo, Norway; 6 Department of Clinical Medicine, The University of Bergen, Jonas Lies vei 65, 5021 Bergen, Norway; 7 National Centre for Ultrasound in Gastroenterology, Haukeland University Hospital, Jonas Lies vei 65, 5021 Bergen, Norway

**Keywords:** sonoporation, microbubbles, ultrasound, intracellular signaling, phosphorylation, ultrasound contrast agents, drug delivery, cellular stress

## Abstract

Treatment with ultrasound and microbubbles (sonoporation) to enhance therapeutic efficacy in cancer therapy is rapidly expanding, but there is still very little consensus as to why it works. Despite the original assumption that pore formation in the cell membrane is responsible for increased uptake of drugs, the molecular mechanisms behind this phenomenon are largely unknown. We treated cancer cells (MOLM-13) and healthy peripheral blood mononuclear cells (PBMCs) with ultrasound at three acoustic intensities (74, 501, 2079 mW/cm^2^) ± microbubbles. We subsequently monitored the intracellular response of a number of key signaling pathways using flow cytometry or western blotting 5 min, 30 min and 2 h post-treatment. This was complemented by studies on uptake of a cell impermeable dye (calcein) and investigations of cell viability (cell count, Hoechst staining and colony forming assay). Ultrasound + microbubbles resulted in both early changes (p38 (Arcsinh ratio at high ultrasound + microbubbles: +0.5), ERK1/2 (+0.7), CREB (+1.3), STAT3 (+0.7) and AKT (+0.5)) and late changes (ribosomal protein S6 (Arcsinh ratio at low ultrasound: +0.6) and eIF2α in protein phosphorylation). Observed changes in protein phosphorylation corresponded to changes in sonoporation efficiency and in viability, predominantly in cancer cells. Sonoporation induced protein phosphorylation in healthy cells was pronounced (p38 (+0.03), ERK1/2 (−0.03), CREB (+0.0), STAT3 (−0.1) and AKT (+0.04) and S6 (+0.2)). This supports the hypothesis that sonoporation may enhance therapeutic efficacy of cancer treatment, without causing damage to healthy cells.

## 1. Introduction

The use of microbubbles (MB), such as ultrasound contrast agents, together with ultrasound (US) to improve therapeutic efficacy has a multitude of applications, ranging from enhancing drug penetration through tissue, opening of the blood brain barrier, to sonothrombolysis [1]. The term “sonoporation” has been commonly used to describe the formation of pores in cells using US + MB, derived from the term “electroporation”, and is commonly used to describe the pore formation phenomenon observed in cell culture experiments. The therapeutic effects of US + MB enhanced therapy have shown great potential over the last 20 years. It can be used to increase the delivery and resulting efficacy of drugs and is therefore particularly useful in cancer therapy, where poor uptake of drugs is one of the factors limiting therapeutic effect [2]. The therapeutic benefits of US + MB enhanced therapy in cancer have been demonstrated in numerous preclinical trials [3,4,5,6]. Furthermore, a Phase I clinical trial [7] showed that US + MB enhanced therapy in combination with chemotherapy is safe and may increase survival of patients suffering from pancreatic ductal adenocarcinoma, and is being followed by clinical trials in other cancers [8,9,10].

Numerous in vitro studies have demonstrated that the combined use of ultrasound and microbubbles forms pores in the membranes of cancer cells [11]. The improved chemotherapeutic efficacy was subsequently assumed to be due to passive diffusion of the drugs through the pores. Nevertheless, despite substantial research in this field, it is not fully understood if the mechanical stresses induced by the microbubbles contribute to additional molecular mechanisms. It has been suggested that cellular stress induced by sonoporation may contribute to the enhancement of cancer therapy [12]. These additional therapeutic effects, which are induced by the biophysical stimuli of the microbubbles interacting with cells, must involve activation of intracellular signaling pathways. However, it is still not known how protein phosphorylation in intracellular signaling pathways changes in response to sonoporation. Low intensity ultrasound used to accelerate bone and joint healing, has been found to induce integrin/mechanotransduction signaling that include proteins like p38, ERK1/2 and JNK (Mitogen-activated protein (MAP)-kinase pathway) and focal adhesion kinase (FAK), in chondrocytes [13] and synovial cells [14]. Additionally, low intensity ultrasound has been described to stimulate cell growth though the integrin/FAK/phosphoinositide-3-kinase-protein kinase (PI3K) pathway, including Akt, in chondrocytes [15] and osteoblasts [16]. As sonoporation also includes the use of microbubbles, knowledge on how addition of microbubbles influences the cellular responses is needed. In respect to the clinical application of this approach, it would further be of great importance to understand how sonoporation influences different cell types. Specifically, to contrast the effects induced on cancer cells or healthy cells, of which both will inevitably be exposed during cancer therapy.

To gain more insight into the molecular mechanisms of sonoporation, our aim was to analyze phosphorylation changes in intracellular signaling networks following treatment with ultrasound with and without the addition of microbubbles. Uptake of a cell impermeable dye and cell viability assays were used to determine the efficacy of sonoporation. We have investigated the intracellular signaling responses of increasing ultrasound intensity and addition of microbubbles using two commercially available ultrasound contrast agents in both a cancer cell line and healthy peripheral blood mononuclear cells (PBMCs).

## 2. Materials and Methods

### 2.1. Chemicals

All chemicals were purchased from Merck KGaA (Darmstadt, Germany) unless otherwise stated.

### 2.2. Microbubbles

Sonazoid^TM^ (GE Healthcare, Little Chalfont, UK) was reconstituted by adding 2 mL NaCl 9 mg/mL (Fresenius Kabi, Bad Homburg vor der Höhe, Germany) and gently agitating for 30 s. The bubbles were aspirated via a 19 G needle, and a 19 G venting needle was used to avoid a pressure drop in the vial. Bubbles were diluted in NaCl 9 mg/mL immediately before addition to cells. SonoVue^®^ (Bracco S.p.A., Milan, Italy) was prepared following manufacturer specifications. To ensure that the reconstituted bubbles were stable, Sonazoid™ bubbles were used within 1 h of reconstitution, and SonoVue^®^ bubbles within 30 min.

### 2.3. Cell Culture

The leukemic cell-line MOLM-13 (DSMZ, Braunschweig, Germany) was cultured in RPMI 1640 medium supplemented with 10% fetal bovine serum and 1% l-glutamine. Peripheral blood mononuclear cells (PBMCs) were isolated from concentrated leukocyte suspensions of healthy donors, generated from whole blood by centrifugation. Blood was supplied from healthy volunteers, from the Department of Immunology at Haukeland University Hospital, Bergen, Norway. PBMCs were isolated using density gradient separation (Lymphoprep^TM^, Abbott Laboratories, Chicago, IL, USA), followed by red blood cell lysis performed in Red Cell lysis buffer (155 mM NH_4_Cl, 10 mM NaHCO_3_, 0.1 mM EDTA and distilled water). PBMCs were cultured in RPMI 1640 supplemented with 10% FBS and 1% l-glutamine. All cells were cultured in a 5% CO_2_ humidified atmosphere at 37 °C.

### 2.4. In Vitro Treatment with Ultrasound and Microbubbles

**Figure 1:**
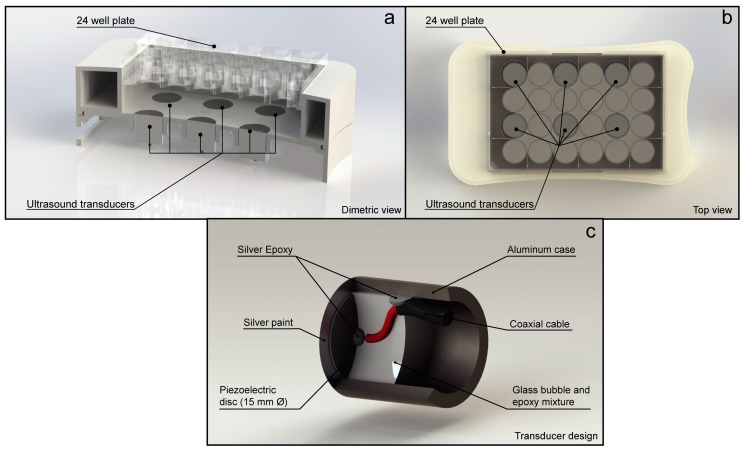
Ultrasound treatment chamber and transducer design used to treat the cells. (**a**) Cutaway of the dimetric view of ultrasound treatment chamber showing the distance. (**b**) Top view of the chamber showing which cell culture wells were exposed to ultrasound. (**c**) Cutaway of the transducer design used in the ultrasound treatment chamber.

MOLM-13 cell suspension was seeded into six wells of a 24-well plate (TPP Techno Plastic Products AG, Trasadingen, Switzerland). A total of 1 × 10^6^ cells/well in 2.5 mL supplemented RPMI 1640 culture media were seeded (0.4 × 10^6^ cells/mL) and rested for approximately 1–2 h prior to sonication. The MOLM-13 cell line was exposed to ultrasound whilst in exponential growth. The PBMCs were treated directly following separation from blood. A total of 8 × 10^6^ cells/well in 2.5 mL supplemented RPMI 1640 culture media (3.2 × 10^6^ cell/mL) were seeded and rested for approximately 30–60 min prior to sonication. As MOLM-13 cells are larger than PBMC cells, the PMBC cell concentration was increased to match the total cell volume of the MOLM-13 experiments (Figure S1). Microbubbles were added at a final concentration of 0.4 × 10^6^ bubbles/mL (1 × 10^6^ bubbles per well), based on an a priori dose-response study (Figure S2). This microbubble dose correlates to a clinical dose of 1.7 mL of Sonazoid^TM^, and 10.0 mL of SonoVue^®^. Both doses are above clinical imaging and diagnostic recommendations from the manufactures. Immediately following bubble addition, a self-adhering membrane (TopSeal™–A, Perkin Elmer, Waltham, MA, USA) was stuck on the top surface of the 24-well plate to create a watertight seal. This membrane was used as the acoustic propagation interface and the acoustic amplitude was compensated for the membrane attenuation (measured to be <1% at 1.0 MHz). The plate was inverted, placed in the ultrasound treatment chamber [17] and ultrasound was applied. Figure 1 shows a schematic of the ultrasound treatment chamber. In short, the ultrasound chamber consisted of 6 single element, unfocused, disc transducers (PZ26, 2 mm thickness, 15 mm diameter). A 3D printed chamber was used to accurately align the transducers and 6 wells within the 24 well plate. The center of the water volume in the cell culture wells was aligned with the axial acoustic focus. Three different US conditions were used (Table 1), referred to as “Low”, “Medium”, or “High”. All samples were sonicated for 10 min. In all experiments the Mechanical Index (MI) was kept below 0.4, which is generally considered safe [18]. Only the “High” acoustic condition was above the clinical diagnostic imaging guidelines in terms of the I_SPTA_ value. Nevertheless, much higher values are already used in therapeutic ultrasound clinical trials.

### 2.5. Uptake of Cell Impermeable Dye

To measure sonoporation efficiency calcein, a non-toxic, cell impermeable fluorescent dye, was added during treatment with ultrasound ± microbubbles. Calcein was dissolved in 1 M NaOH to a stock solution of 50 mg/mL and protected from light at 2–8 °C. For each experiment, a fresh solution of 2.5 mg/mL in 9 mg/mL NaCl (Fresenius Kabi, Bad Homburg vor der Höhe, Germany) was prepared. Calcein was added to the cell suspension to a concentration of 5 µM immediately before sonication. After ultrasound treatment, the cells were incubated for 40–60 min, washed twice in PBS, and subsequently analyzed using an Accuri C6 flow cytometer (BDBioscience, Franklin Lakes, NJ, USA). Incubation time was evaluated prior to experiments (Figure S3). A duration of 40 min or more was required due experimental feasibility constraints. An incubation time of 60 min was chosen as this was an indicated pore re-sealing time [19]. A minimum of three replicates were analyzed per experiment, and all experiments were performed three times.

### 2.6. Viability Analysis

In separate experiments without calcein, cells were treated with ultrasound ± microbubbles and samples were harvested for both viability assays and studies on intracellular signaling. Cell concentration was determined by manual counting using a haemocytometer. This was done immediately after treatment with ultrasound and after 24 h of culture. Apoptotic cell death was assessed by adding 0.01 mg/mL Hoechst 33342 (Merck KGaA, Darmstadt, Germany) to cells after 24 h of culturing. Cells were fixed in 4% formaldehyde and imaged by fluorescence microscopy. The total number of cells was determined in Fiji [20] by adjusting the image brightness and contrast followed by a manual threshold then using the “Analyze particles” function. Apoptotic cells were counted manually using the “Cell counter” plugin in Fiji. Colony forming assay (Methocult™ Classic H4434; Stemcell™ Technologies, Vancouver, BC, Canada) was used to evaluate the proliferation of the MOLM-13 cells. Cells were seeded immediately after treatment and colonies were counted after 7–10 days incubation. All experiments were performed in triplicate.

### 2.7. Sample Preparation for Phosphospecific Flow Cytometry

Samples for intracellular signaling studies were taken at three different time points: immediately after sonoporation (approximately 5 min), after 30 min, and 2 h of incubation. Cells were fixed by adding 16% paraformaldehyde (Alfa Aesar, Haverhill, MA, USA) to a final concentration of 2% and incubating for 15 min at room temperature, washed in cold PBS and permeabilized in ice-cold methanol, following a modified protocol [21,22]. As positive controls for intracellular signaling, MOLM-13 cells were treated with either 1 μM A23187 (Calcimycin; calcium ionophore) for 4 h, 100 ng/mL TNF-α for 15 min, or 1 μM A23187 + 100 nM phorbol myristate acetate (PMA; PKC activator) for 30 min. PBMCs were treated with 1 μM A23187 + 100 nM PMA for 30 min as positive control. Variation in response to A23187 + PMA between cells from 3 different donors and MOLM-13 are shown in Figure S6.

### 2.8. Barcoding and Antibody Staining

Fluorescent cell barcoding was used to perform multiplex flow cytometry. Individual cell samples were stained with unique signatures of succimidylesters of Pacific Blue (0.05 μg/mL, 0.5 μg/mL, 5 μg/mL) and Pacific Orange (0 μg/mL, 2.5 μg/mL and 20 μg/mL). After barcode staining, the samples were pooled prior to antibody staining [23]. A graphical depiction of barcoding/sample preparation for phospho-flow cytometry is shown in Figure S4. One barcode represents all samples from one timepoint in each experiment. Barcoded cells were split into staining panels and each panel was stained separately with a combination of antibodies conjugated to either Alexa Fluor^®^ 488 or 647 (Table S1). PBMC samples were incubated with FcR-blocking reagent (Miltenyi Biotec, Bergisch Gladbach, Germany) prior to antibody staining. Samples were analyzed on an LSR Fortessa flow cytometer (BDBioscience, Franklin Lakes, NJ, USA). After flow cytometric analysis the samples were de-barcoded and the arcinhratio for each treated sample to the untreated cells, harvested at the same timepoint, was calculated.

### 2.9. Western Blots

Samples for western blot were harvested 2 h post sonoporation, washed twice in PBS and lysed in RIPA buffer containing protease- and phosphatase inhibitor (Thermo Fisher Scientific, Waltham, MA, USA). Protein concentration was quantified in accordance with Bio-Rad DC protein assay instruction manual for microtiter plates. For the western blots 10% SDS-polyacrylamide gels were loaded with 20–30 μg protein per well. Proteins were separated at 100–120 V and transferred to a nitrocellulose membrane (240 mA, 150 min, 4 °C), followed by 1 h blocking using 5% skim milk powder in Tris-buffered saline (TBS) + 1% Tween 20. Primary antibodies were diluted in 5% skim milk in TBS-Tween and incubated over night at 4 °C. Primary antibodies used were p-eIF2α Ser-51 (Abcam, Cambridge, UK) and eIF2α (Abcam). COX IV (Abcam) was used as loading control. Secondary antibodies were diluted in 5% skim milk powder in TBS-Tween 20 and incubated for 1 h at room temperature. Membranes were developed using SuperSignal^®^ West Pico or SuperSignal^®^ West Femto Chemiluminescence Substrate (Thermo Fischer Scientific) in accordance with manufacturers recommendations.

### 2.10. Data Analysis/Statistical Analysis

Data collected from the Accuri C6 flow cytometer was analyzed in FlowJo^®^ (BDBioscience, Franklin Lakes, NJ, USA). Data collected from LSR Fortessa on barcoded samples were de-barcoded and gated in FlowJo^®^. Gating strategy is shown in Supplemental Figure S5. Further analysis was performed in Cytobank (Cytobank Inc., Santa Clara, CA, USA). Arcsinh ratio (arcsinh(treated/5)- arcsinh(control/5)) was used to calculate changes in phosphorylation. Statistical comparisons were performed in Prism 6 (GraphPad Software, San Diego, CA, USA) using unpaired two-tailed *t*-tests. Significance level was set at *p*-value 0.05. All *p*-values are shown in Tables S2–S6.

## 3. Results

### 3.1. Efficiency of Sonoporation Was Increased by Addition of Microbubbles and High Ultrasound Intensity

#### 3.1.1. Percentage of Cells Taking Up Calcein (Permeabilization Efficiency)

**Figure 2:**
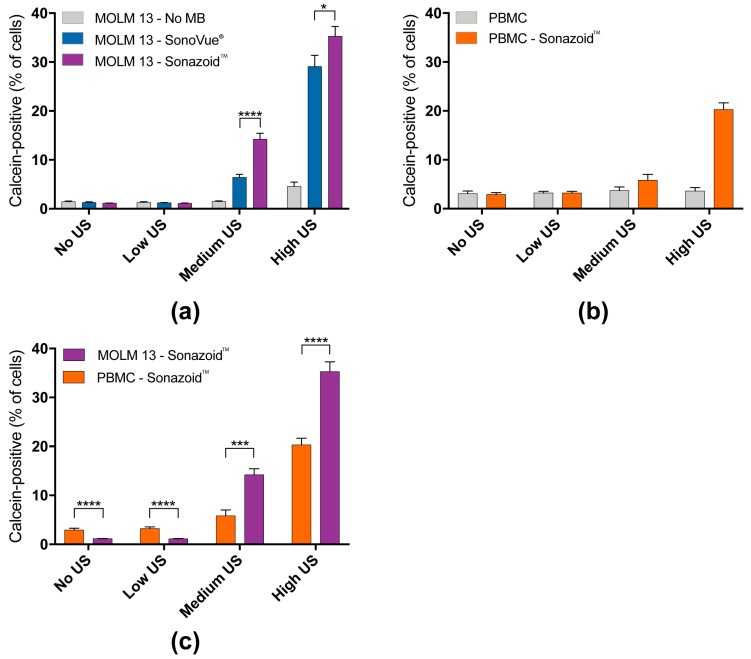
Increasing the ultrasound intensity and the addition of microbubbles significantly increased the percentage of calcein-positive cells. (**a**) Percentage of calcein-positive MOLM-13 cells versus ultrasound intensity. Increasing the ultrasound intensity correlated to an increase in calcein positive cells. The addition of microbubbles significantly increased the calcein-positive population. At medium and high ultrasound intensities Sonazoid™ was significantly better than SonoVue^®^. (**b**) Percentage of calcein positive peripheral blood mononuclear cells (PBMCs) versus ultrasound intensity. Ultrasound alone did not increase the calcein-positive population. The addition of Sonazoid™ significantly increased the calcein-positive population at medium and high ultrasound intensities. (**c**) Comparison of calcein-positive population between MOLM-13 cells and PBMCs. At medium and high ultrasound intensities a significantly larger population of the MOLM-13 cells were calcein positive. * = *p* < 0.05, ** = *p* < 0.01, *** = *p* < 0.001, **** = *p* < 0.0001 (Significance depicted for Sonazoid™ vs. SonoVue^®^, and MOLM-13 vs. PBMC. All *p*-values can be found in Tables S2–S5).

We investigated sonoporation efficiency by measuring uptake of the cell impermeable dye calcein into the cells, a measure of cell permeabilization. Figure 2 shows the percentage of cells positive for calcein after ultrasound treatment ± microbubbles. Increasing ultrasound intensity increased the percentage of calcein-positive cells (Figure 2a,b). In the absence of microbubbles, only high ultrasound intensity induced a significant increase in calcein-positive MOLM-13 cells. The addition of either SonoVue^®^ or Sonazoid™ microbubbles resulted in a significantly increased number of calcein-positive cells at medium or high ultrasound intensity (No bubbles: 1.5% and 1.5%; SonoVue^®^: 6.4% and 29%; Sonazoid™: 14% and 35%, at medium and high ultrasound respectively) (Figure 2a, Tables S2 and S3). Sonazoid™ induced a significantly higher percentage of calcein-positive cells than SonoVue^®^ (Table S4) and based on these results we used Sonazoid™ for subsequent experiments. In PBMCs, the addition of Sonazoid™ increased the number of calcein-positive cells to 6% at medium ultrasound and to 20% at high ultrasound intensity, while no significant difference was observed without the use of microbubbles (Figure 2b). Figure 2c compares the efficacy of each ultrasound condition + Sonazoid™ for MOLM-13 cells and PBMCs. At medium and high ultrasound intensities, a significantly larger population of the MOLM-13 cells vs. PBMCs were calcein-positive (Table S5).

#### 3.1.2. Quantified Uptake of Calcein

**Figure 3:**
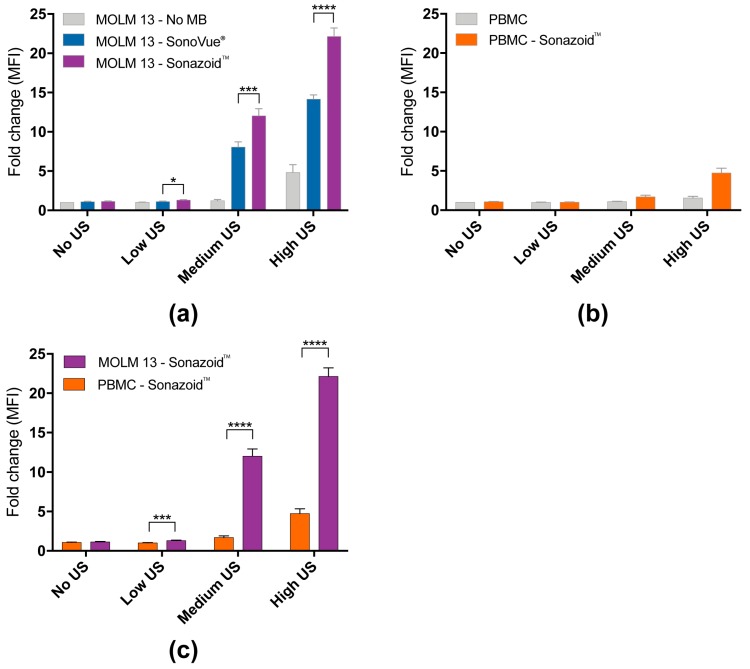
Sonoporation induced significant increase in uptake of model drug calcein in sonoporated cells measured by median fluorescence intensity. (**a**) Fold change of median fluorescence intensity (MFI) in MOLM-13 cells affected by sonoporation (=calcein-positive cells). The increase in calcein uptake depended on addition of microbubbles and ultrasound intensity. Use of Sonazoid resulted in significantly higher increase in uptake than SonoVue at all three ultrasound intensities. (**b**) Fold change of median fluorescence intensity (MFI) in calcein-positive PBMC. The uptake is significantly dependent on addition of microbubbles and ultrasound intensity (significant increase in uptake only seen at high ultrasound intensity). (**c**) Comparison of calcein uptake between MOLM-13 and PBMC. A significantly higher percentage of cancerous MOLM-13 cells were affected than healthy PBMC at all 3 ultrasound intensities. * *p* < 0.05, ** *p* < 0.01, *** *p* < 0.001, **** *p* < 0.0001 (Significance depicted for Sonazoid™ vs. SonoVue^®^, and MOLM-13 vs. PBMC. All *p*-values can be found in Tables S2–S5).

The median fluorescence intensity (MFI) was used as an estimate of calcein uptake and is depicted as fold change of controls not treated with ultrasound or microbubbles (Figure 3). As for the percentage of calcein-positive cells (Figure 2c) the effect of increasing ultrasound intensity alone was only significant at high ultrasound intensity, while the addition of microbubbles increased calcein uptake at all ultrasound intensities in MOLM-13 cells (Figure 3a, Tables S2 and S3). The uptake enhancement by using Sonazoid™ vs. SonoVue^®^ was confirmed (SonoVue^®^: 8-fold and 14-fold increase at increase at medium and high ultrasound intensity respectively; Sonazoid™: 12-fold and 22-fold) at all three ultrasound intensities (Table S4). In PBMCs a significant increase in calcein uptake was observed at medium and high ultrasound intensity + Sonazoid™ (Figure 3b). The uptake was significantly higher in MOLM-13 cells than PBMCs at all 3 ultrasound intensities (Figure 3c, Table S5).

### 3.2. Decreased Viability in Cancerous MOLM-13 Cells after Sonoporation

**Figure 4:**
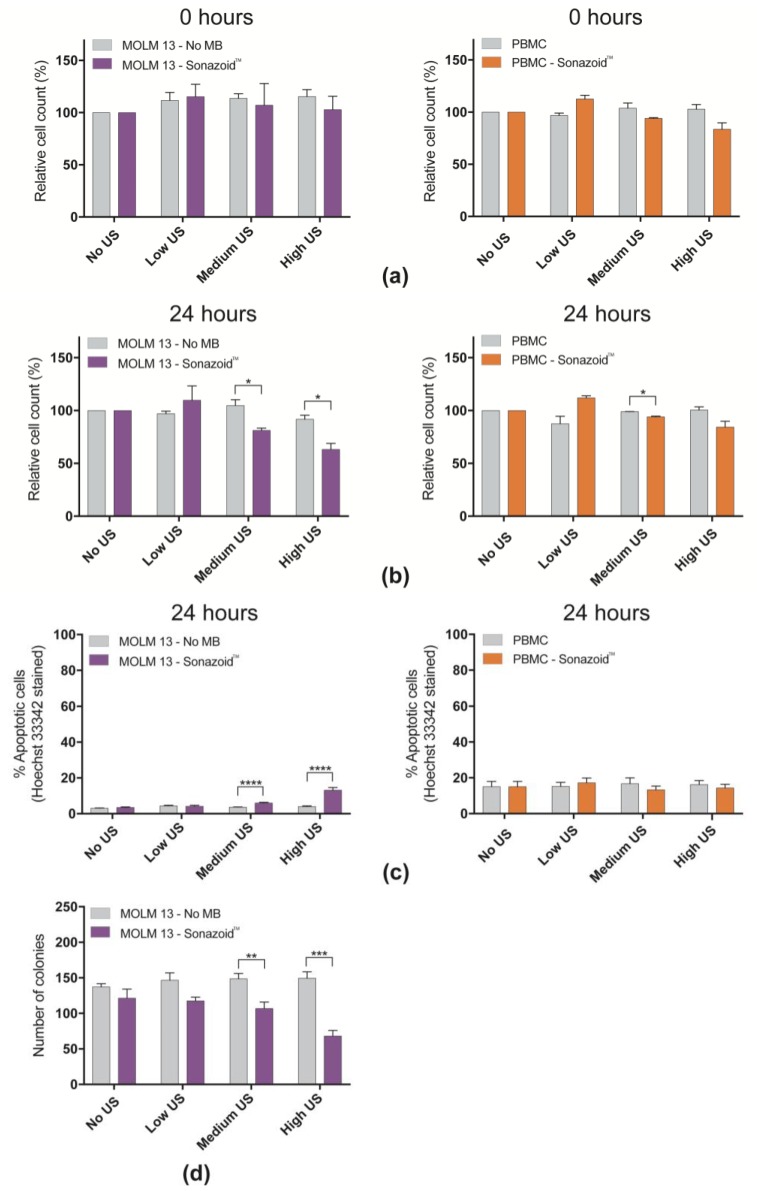
Viability of cells in response to ultrasound ± Sonazoid^TM^ microbubbles (**a**) Normalized cell count at different ultrasound intensities ± Sonazoid^TM^ microbubbles (normalized to cells not treated with ultrasound), of cells harvested immediately after sonoporation (0 h). No significant change in cell count due to sonoporation was observed (**b**) Normalized cell count of cells harvested 24 h after sonoporation. After 24 h the cell count was significantly lower in samples treated with medium (*p* < 0.05) and high (*p* < 0.05) ultrasound intensity. The cell count of PBMCs did not change much after 24 h in any of the samples. A small increase in cell count was observed in the samples treated with the lowest ultrasound intensity, but this effect is not significant. A small, but significant, decrease in cell count was observed in the sample treated with medium ultrasound intensity. (**c**) Apoptotic cells 24 h post sonoporation by Hoechst 33342 staining in MOLM-13 and PBMC (**d**) Number of colonies of MOLM-13 formed post sonoporation (colony forming assay). * *p* < 0.05, ** *p* < 0.01, *** *p* < 0.001, **** *p* < 0.001.

The cell count was measured immediately after sonoporation to determine if the mechanical forces lead to any immediate cell damages from the treatment. Cell lysis or immediate necrosis was only observed at much higher acoustic intensities than those evaluated in this study, (i.e., MI = 0.6, I_SPTA_ = 2673mW/cm^2^, Duty Cycle = 20%). No significant changes in total cell counts were observed for either MOLM-13 or PBMCs at any of the ultrasound intensities when compared to controls not treated with ultrasound (Figure 4a,d, Tables S2 and S3). However, a small increase in cell count was observed using low ultrasound without microbubbles at both 0 and 24 h compared to untreated cells. After 24 h a significantly lower cell count was observed in MOLM-13 cells treated with medium and high ultrasound with the addition of microbubbles (Figure 4b, Tables S2 and S3). In PBMCs a modest, but statistically significant, decrease in cell count after 24 h was observed at medium ultrasound with microbubbles (*p* < 0.05) (94% and 99% cells relative to untreated cells, respectively). To elucidate if the reduced cell count of MOLM-13 was a result of increased cell death or reduced proliferative ability, Hoechst 33342 staining and colony forming assay were performed. The addition of microbubbles and application of medium or high US increased the percentage of apoptotic MOLM-13 cells to 6% and 13% respectively (Figure 4c, Tables S2 and S3). Upon Hoechst staining of PBMCs, no significant change in apoptotic cells was observed at any treatment regime. Colony forming assays (Figure 4d, Tables S2 and S3) demonstrated that MOLM-13 cells formed significantly less colonies at medium and high ultrasound intensity + microbubbles (77% and 50% fewer colonies, respectively).

### 3.3. Sonoporation Induced Changes in Intracellular Signaling-Profiles

**Figure 5:**
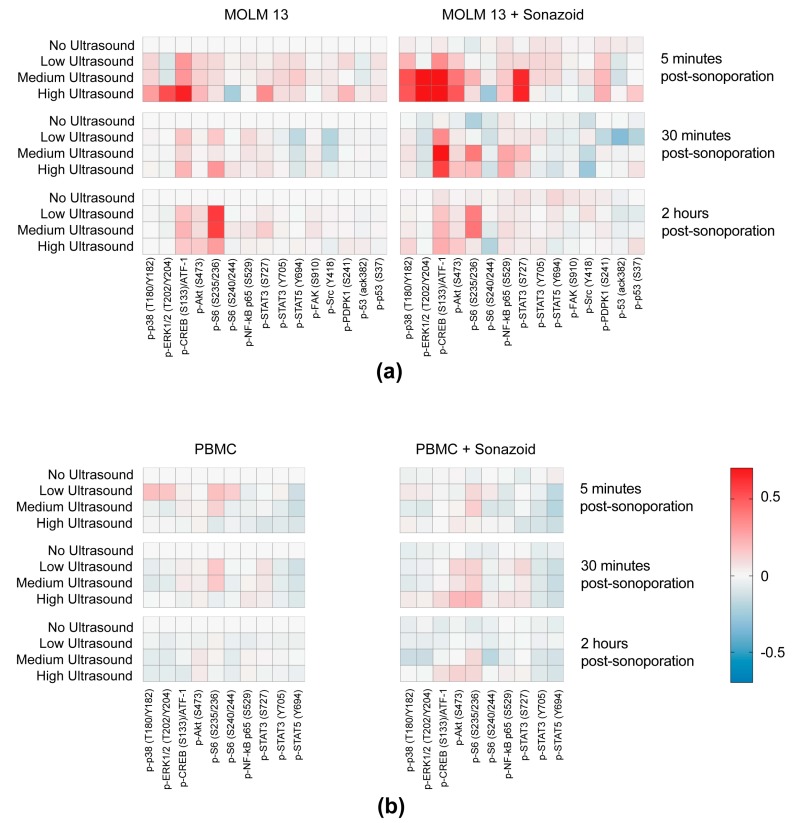
Intracellular signaling profiles of sonoporated cells. (**a**) Heatmaps displaying changes in phosphorylation status in MOLM-13 of the chosen range of proteins in response to treatment with ultrasound with and without Sonazoid^TM^ microbubbles. Phosphorylation status was detected 5 min, 30 min and 2 h post sonoporation (mean of Arcsinh ratios) (**b**) Heatmaps displaying changes in phosphorylation status in PBMC of the chosen range of proteins in response to treatment with ultrasound with and without Sonazoid^TM^ microbubbles. Phosphorylation status was detected 5 min, 30 min and 2 h post sonoporation (mean of Arcsinh ratios).

In line with the results observed from calcein uptake experiments (Figure 2 and Figure 3) the changes in intracellular signaling were more pronounced in MOLM-13 cells (Figure 5a) compared to PBMCs (Figure 5b). Significantly increased signaling was observed at medium and high ultrasound intensity and when microbubbles were added during sonication (Figure 5a, Table S6). Increased phosphorylation from untreated cells was observed for p-38 T180/Y182, ERK1/2 T202/Y204, CREB S133/ATF-1, Akt S473 and STAT3 S727 in the MOLM-13 cell line. Sonoporation altered STAT3 phosphorylation specifically at the Ser727 epitope, and there was no change in phosphorylation status on the Tyr 705 epitope. STAT5 phosphorylation level was not affected by sonoporation. Phosphorylation status of FAK, NF-kB, Src, PDPK1 or p53 did not change.

#### 3.3.1. Immediate Effects of Sonoporation

**Figure 6:**
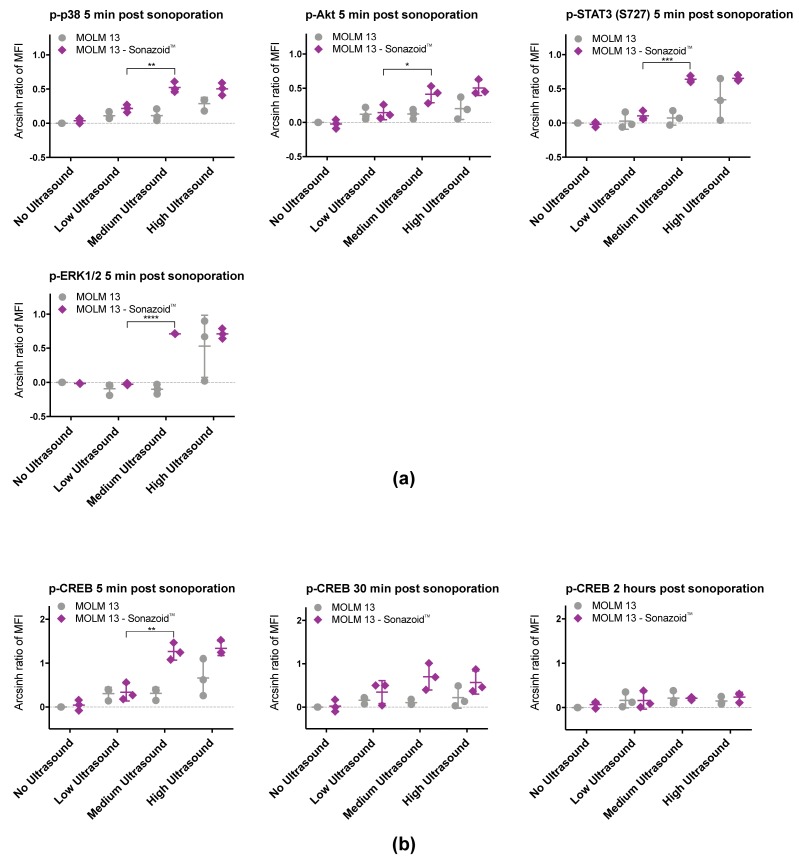
Significant changes in phosphorylation were observed for p38, ERK1/2, CREB, Akt and STAT3 (S727) in MOLM-13 cells. The increases in phosphorylation primarily occurred when increasing ultrasound from low to medium. (**a**) Scatter plots of results 5 min post sonoporation from 3 individual experiments. Phosphorylation status of p38, ERK1/2, Akt and STAT3 (S727) were transiently altered from untreated controls at medium and high ultrasound intensity in the presence of microbubbles. (**b**) Scatter plots of results at 5 min, 30 min and 2 h from 3 individual experiments. Phosphorylation status of CREB was altered by sonoporation for up to 2 h. * *p* < 0.05, ** *p* < 0.01, *** *p* < 0.001, **** *p* < 0.001 (Significance depicted for Low ultrasound vs. Medium ultrasound. All *p*-values can be found in Tables S2–S5).

Changes in intracellular signaling by sonoporation in MOLM-13 were mostly observed immediately after treatment (Figure 5a). Phosphorylation of p38 T180/Y182, ERK1/2 T202/Y204, Akt S473, and STAT3 S727 (Figure 6a, Table S5) was significantly increased 5 min after sonoporation. Phosphorylation generally decayed rapidly and the difference from controls was either lower or not detected 30 min after sonoporation. Changes in phosphorylation were primarily observed when cells were treated with medium and high ultrasound intensity with microbubbles. Only phosphorylation of p38 was significantly increased without microbubbles, and only at high ultrasound intensity. A significant increase in phosphorylation immediately after sonoporation with microbubbles was also observed for CREB S133/ATF-1. However, this was sustained for a longer time period. Phosphorylation of CREB S133/ATF-1 was significant increased for up to 2 h when using medium or high US + MB (Figure 6b, Table S5). Furthermore, the changes in phosphorylation of p38 T180/Y182, ERK1/2 T202/Y204, CREB S133/ATF-1, Akt S473 and STAT3 S727 are predominantly seen when increasing ultrasound from low to medium intensity (+ microbubbles). No significant differences were found when increasing from medium to high ultrasound intensity (Figure 6, Table S6).

#### 3.3.2. Downstream Effects of Sonoporation—Ribosomal Protein S6

**Figure 7:**
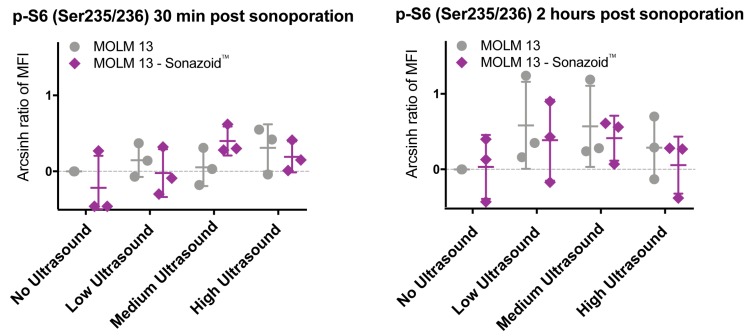
Changes in phosphorylation as observed for ribosomal protein S6 in MOLM-13 cells, shown as scatter plots of results at 30 min and 2 h from three individual experiments. The trend is an increase in phosphorylation at low and medium ultrasound intensity both with and without microbubbles, which is reduced at high ultrasound intensity. Changes in phosphorylation of ribosomal protein S6 at Ser 235/236 were not significant. This is probably due to high variation in results between experiments.

Not all the effects of sonoporation on intracellular signaling were observed immediately after treatment. A difference in phosphorylation status of ribosomal protein S6, specifically on the S235/236 epitope, was seen 2 h after sonoporation (Figure 7). At high ultrasound, phosphorylation was lower than at the lower ultrasound intensities and in particular at high ultrasound with addition of microbubbles there is almost no difference in phosphorylation compared to untreated controls. At low and medium ultrasound intensity the phosphorylation is higher than untreated controls both with and without microbubbles. A similar yet very weak response was also detected in PBMCs (Figure 5b). However, none of the observed changes in phosphorylation of ribosomal protein S6 were statistically significant. No changes from untreated controls in phosphorylation were observed at the S240 epitope on ribosomal protein S6.

#### 3.3.3. Downstream Effects of Sonoporation—Eukaryotic Initiation Factor 2α

**Figure 8:**
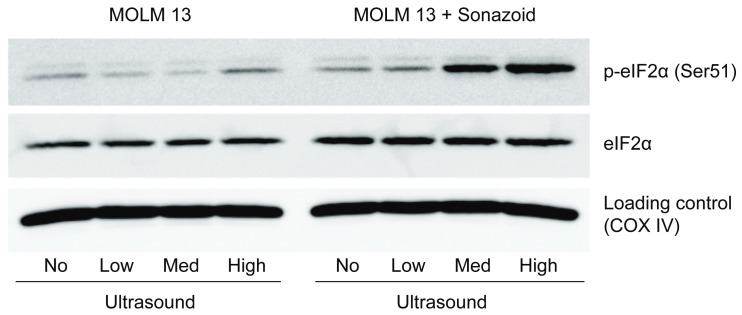
Phosphorylation of eIF2α in MOLM-13 (Western blot) when treated with medium and high ultrasound in the presence of microbubbles 2 h post sonoporation.

Eukaryotic initiation factor 2 alpha (eIF2α) signaling may have an inverse relationship to mTOR/S6 signaling [24], and to elucidate the downstream mechanism the phosphorylation status of eIF2α was investigated. The Western blot in Figure 8 shows that eIF2α was phosphorylated 2 h post sonoporation in MOLM-13 when exposed to medium and high ultrasound intensity with addition of microbubbles. The phosphorylation of eIF2α was increasing from medium to high ultrasound (+ microbubbles).

## 4. Discussion

In this study we present a new insight in the cellular responses following sonoporation, by an extensive screen of phosphorylation changes in intracellular signaling pathways. Although a range of bioeffects in response to sonoporation is already known [25,26], the intracellular signaling responses have so far been overlooked. We found that sonoporation activates key signaling pathways, such as the MAP-kinase pathway and PI3K-pathway, and both transcription and translation factors. The use of our high-throughput ultrasound treatment chamber [17] enabled us to relate these new findings with more commonly used measures of sonoporation efficiency: uptake of a cell-impermeable dye [19,27,28,29,30,31] (permeabilization) and cell viability [19,27,31,32,33]. Interestingly, there is a shift in the phosphorylation profile of the cells at the acoustic/microbubble conditions in which calcein uptake is increased and viability is decreased, and this trend corresponds with increasing ultrasound intensity and was significantly enhanced by addition of microbubbles during US treatment. The results on calcein uptake and viability are in agreement with the existing literature, although an important observation is the higher sensitivity to sonoporation of the cancerous MOLM-13 compared to healthy PBMCs. Also, in the PBMCs, only minor changes in intracellular signaling are detected. The increased susceptibility of cancer cells to ultrasound has previously been observed [34,35], and suggested to be a result of different cell size, morphology or faster growth rate of cancer cells. While the MOLM-13 cells used in this study are indeed larger in size than PBMCs (Figure S1) further work will be required to verify this hypothesis.

Our calcein uptake experiments indicated that Sonazoid™ microbubbles are more effective than SonoVue^®^ microbubbles at the chosen treatment conditions. Whilst both agents are lipid-shell based they have different responses to ultrasound (e.g., different resonant frequencies), gas content, shell stiffness, stability, size distribution, and polydispersity [36,37]. The improved effectiveness of Sonazoid™ could be due to any of these physicochemical properties or behaviors at the chosen ultrasound parameters and concentrations. Changing the acoustic frequencies, concentration, or selecting a specific bubble size population could result in SonoVue^®^ or any other microbubble being more effective.

The effect of sonoporation on intracellular phosphorylation patterns in MOLM-13 can be divided into immediate effects and more long-lasting effects. An immediate and transient increase of phosphorylation was observed for proteins of the MAP-Kinase (ERK, p38) and PI3K (Akt) pathway. Activation of p38, ERK and Akt by ultrasound alone was also observed in previous studies in other cell types [13,14,16]. In contrast to our results, these previous studies showed increased cell growth. The activation of these proteins is known to be involved in increased proliferation of cells, but the broad range of effects of those proteins is not only limited to the regulation of cell growth and differentiation. They also involve transmission of stress stimuli (p38 [38]), cell cycle regulation and proliferation (ERK [39]) and cell survival (Akt [40]). We further observe an increase in phosphorylation of transcription factors CREB and STAT3, which may be activated downstream of MAP-kinases [41,42,43]. For STAT3, this is supported by the fact that it is phosphorylated on the serine 727 epitope only, and there is no change in phosphorylation status on the Tyr705 epitope which is phosphorylated downstream of JAK in the JAK/STAT pathway [41,42].

The downstream effects of sonoporation in MOLM-13 that we have observed are changes in phosphorylation of eIF2α and ribosomal protein S6. The phosphorylation of ribosomal protein S6, specifically at the Ser235/236 epitope, followed the inverse trend of immediate signaling events, and was induced by low and medium ultrasound ± microbubbles. This activation was not observed when high ultrasound + microbubbles was used. Ribosomal protein S6 is linked to an increase in cell growth whilst phosphorylation of eIF2α may reduce cell growth [24]. Both eIF2α and ribosomal protein S6 are involved in protein translation, the final step in expression of genes into proteins and a part of biosynthesis that is tightly connected to cell growth. Therefore, these signaling events are in line with our observed decrease in viability at the higher ultrasound plus microbubble conditions. Phosphorylation of eIF2α inhibit general protein translation and cell proliferation in response to cellular stresses, among them endoplasmatic reticulum (ER-) stress—a known bioeffect in response to sonoporation [33]. Activation of ribosomal protein S6 Ser235/236, on the other hand, may simulate cap-dependent protein translation, downstream of ERK [44]. The observed signaling events suggest that sonoporation may influence protein translation.

These results, performed on the same validated experimental platform, indicate that sonoporation may in fact induce both signals, promoting and decreasing viability of cells, depending on ultrasound intensity and the addition of microbubbles. In clinical use, ultrasound parameters, microbubbles and drugs must be chosen carefully, and protein phosphorylation can potentially be used as biomarkers to guide the choice of therapeutic parameters. Furthermore, a reason that the sonoporation mechanisms are still debated is the diversity of experimental parameters, experimental systems and cell types used in the in vitro studies [25,45]. This study also highlights that differences in ultrasound parameters and treatment of different cell types may result in variable, maybe even opposing, biological responses.

Throughout this study, an increase in acoustic intensity induced increased responses. This increased intensity was achieved by increasing both the number of acoustic cycles, i.e., the duty cycle, and peak-negative acoustic pressure, which makes it impossible to correlate the effects specifically to an increase in either MI or duty cycle. Due to thermal and design limitations in the experimental setup, it was not possible to use a single MI value or duty cycle to achieve the given range of acoustic intensities. Further work should be performed to determine which of the two parameters is more critical. Nevertheless, taking into account that microbubble stability is very low after 10–20 continuous ultrasound cycles, we can conjecture that the MI is the dominant factor and a similar response could be observed at clinically relevant acoustic intensities.

Whilst the results in this study signify that each cell type may respond differently to the same treatment conditions, it is important to note that this work was performed in an in vitro configuration that does not accurately mimic the human physiology. Specifically, this experimental configuration used hard plastics that results in acoustic aberrations, there was no flow or temperature and gas saturation regulation, the treatment fluids had a different viscosity to blood, and they were performed in a large open chamber instead of vessel mimicking setup. To validate the results, these experiments should be repeated in a more physiologically relevant model. It should also be noted that even though ultrasound enhanced therapies has been suggested for leukemia [46], the main focus is on solid tumors and the repeated experiments should be performed using cell types constituting solid tumors.

## 5. Conclusions

Our results show that the treatment with ultrasound and microbubbles alone affect viability and intracellular signaling in cells, and this may be relevant for the efficacy of sonoporation to enhance cancer therapy. The cancerous MOLM-13 cells were more susceptible to sonoporation when compared to the healthy blood cells. Ultrasound alone at clinical diagnostic conditions had no significant effect on cell permeabilization, cell viability, or intracellular protein phosphorylation. The addition of microbubbles, or surpassing clinical diagnostic intensities resulted in increased permeabilization efficiency, reduced cell viability and a change in phosphorylation status of p38, ERK1/2, CREB, Akt, STAT3, ribosomal protein S6 and eIF2α in the cancer cells.

## Figures and Tables

**Table 1:** Ultrasound conditions used to treat the cells.

Name	Frequency (MHz)	No. of Cycles	Duty Cycle (%)	Pulse Repetition Frequency (kHz)	MI	Intensity	
						I_SPTA_ (mW/cm^2^)	I_SPPA_ (W/cm^2^)
Low	1.108	4	4	10	0.2	74	0.66
Medium	1.108	18	16	10	0.3	501	2.31
High	1.108	41	37	10	0.4	2079	5.0

## Supplementary Materials

The following are available online at https://www.mdpi.com/1999-4923/11/7/319/s1, Figure S1: Volume normalisation of cell concentration. Figure S2: (a) Percentage calcein-positive cells by increasing concentration of SonazoidTM (b) Calcein uptake (fold change of mean fluorescence intensity) of cells by increasing concentration of SonazoidTM. Figure S3: Optimisation of incubation time post treatment (*n* = 1). Increasing the incubation time results in minor increases in the number of calcein positive cells. Figure S4: Sample processing for phospho-flow cytometry. After sonoporation cells were fixed and permeabilised, barcoded, stained with phosphorylation specific antibodies and analysed by flow cytometry. Barcoding of cells allows for reduced analytical variation. Samples harvested after 5 min, 30 min and 2 h after sonoporation at low medium and high ultrasound intensity were barcoded and pooled in to one sample per timepoint. All treated samples were normalized to untreated cells in the same barcode. Pooled samples were divided in tubes for antibody staining with panels consisting of 2 phosphospecific antibodies conjugated to respectively Alexa 488 and Alexa 647. Figure S5: Gating strategy of flow cytometric data. After identification of the populations the median fluorescence intensity of each sample of cells treated with ultrasound ± microbubbles was compared to the median fluorescence intensity of the untreated cells. Figure S6: Change in phosphorylation status in response to positive controls (A23187 + PMA), represented as arcinhratio normalised to untreated cells. Some variation in response is observed between peripheral blood mononuclear cells (PBMC) from 3 different donors. PBMCs exhibit a stronger reponse to this stimuli compared to cell line MOLM-13. Table S1: Phospho-proteins investigated using flow cytometry. Table S2: The effect of increasing ultrasound intensity on uptake of calcein and viability (Ultrasound treatment vs untreated sample). Table S3: The effect of adding microbubbles on uptake of calcein and viability (Treatment without microbubbles vs treatment with microbubbles). Table S4: The effect of using different bubbles on calcein uptake (SonoVue^®^ vs Sonazoid^TM^). Table S5: The different response in different cell types on calcein uptake (MOLM-13 vs PBMCs). Table S6: The effect of increasing ultrasound intensity and microbubbles on protein phosphorylation (Ultrasound treatment vs untreated sample). Table S7: The effect of increasing ultrasound intensity protein phosphorylation (Ultrasound treatment X vs Ultrasound treatment Y).
