# Supplementary material for: Intracellular Signaling in Key Pathways Is Induced by Treatment with Ultrasound and Microbubbles in a Leukemia Cell Line, but Not in Healthy Peripheral Blood Mononuclear Cells
Source: Pharmaceutics. 2019 Jul 6;11(7):319. doi: 10.3390/pharmaceutics11070319 (PMC6680714; doi:10.3390/pharmaceutics11070319)
Supplement: Supplementary file 1 [file pharmaceutics-11-00319-s001.pdf]

pharmaceutics

Supplementary Materials: Intracellular Signaling in Key Pathways Is Induced by Treatment with Ultrasound and Microbubbles in a Leukemia Cell Line, but not in Healthy Peripheral Blood Mononuclear Cells

Ragnhild Haugse Anika Langer, Stein-Erik Gullaksen, Silje Maria Sundøy, Bjørn Tore Gjertsen, Spiros Kotopoulis and Emmet McCormack

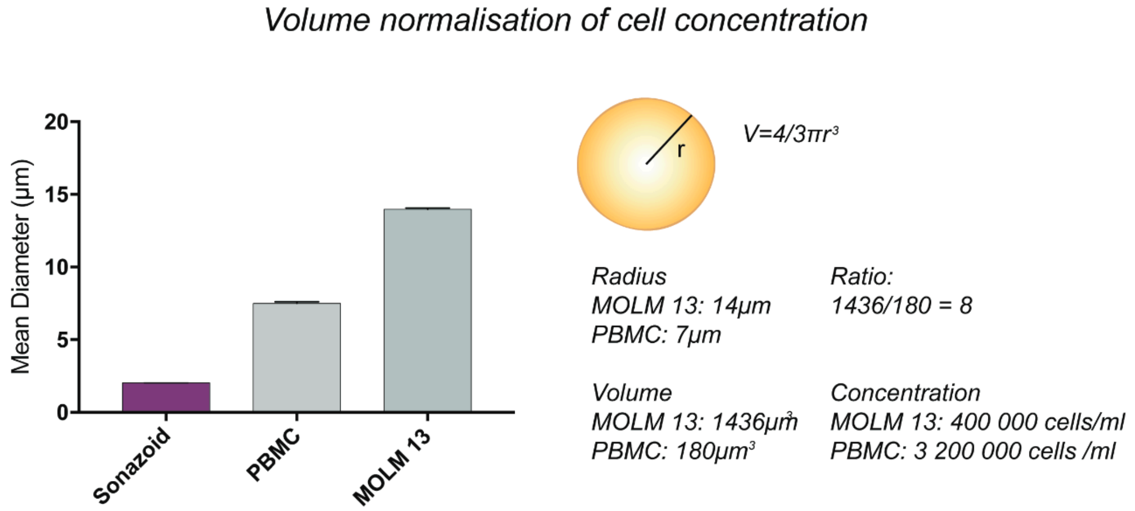

Figure S1. Volume normalisation of cell concentration.

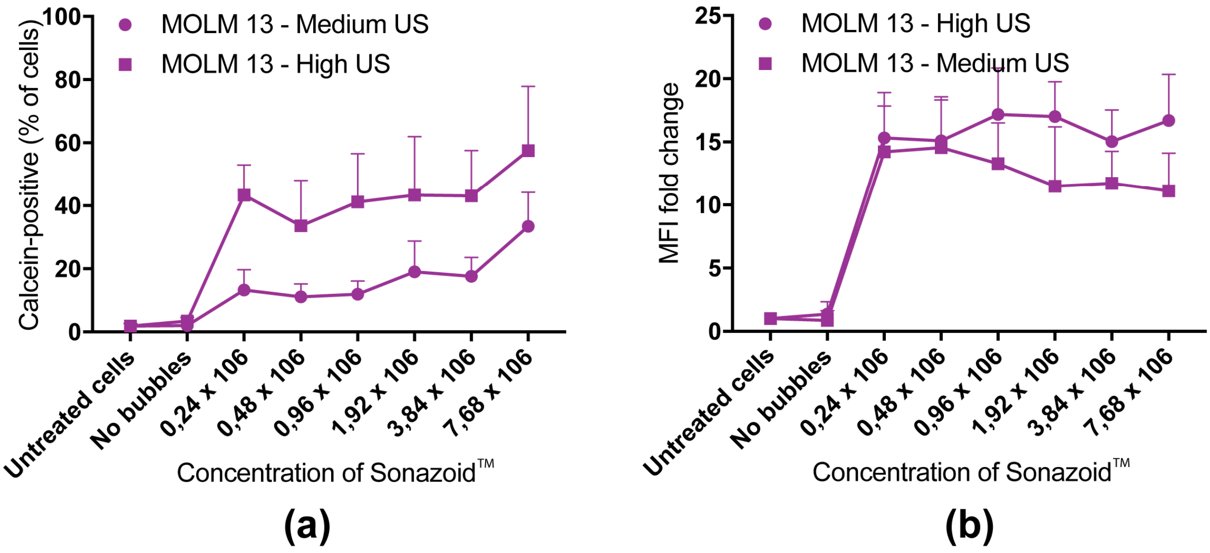

Figure S2. (**a**) Percentage calcein-positive cells by increasing concentration of Sonazoid™ (**b**) Calcein uptake (fold change of mean fluorescence intensity) of cells by increasing concentration of Sonazoid™.

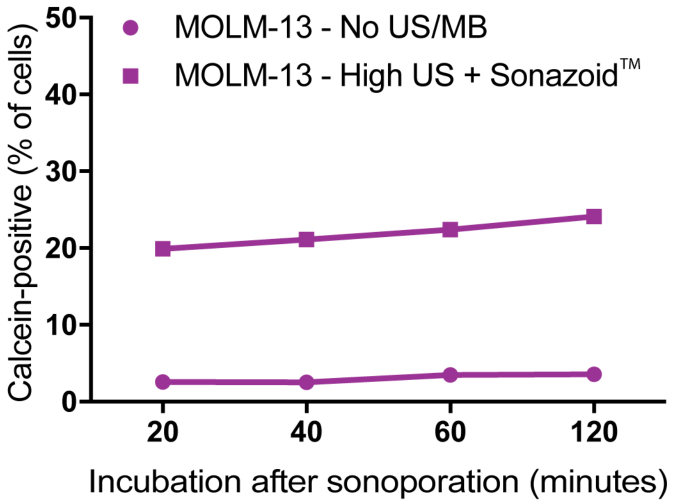

Figure S3. Optimisation of incubation time post treatment (n = 1). Increasing the incubation time results in minor increases in the number of calcein positive cells.

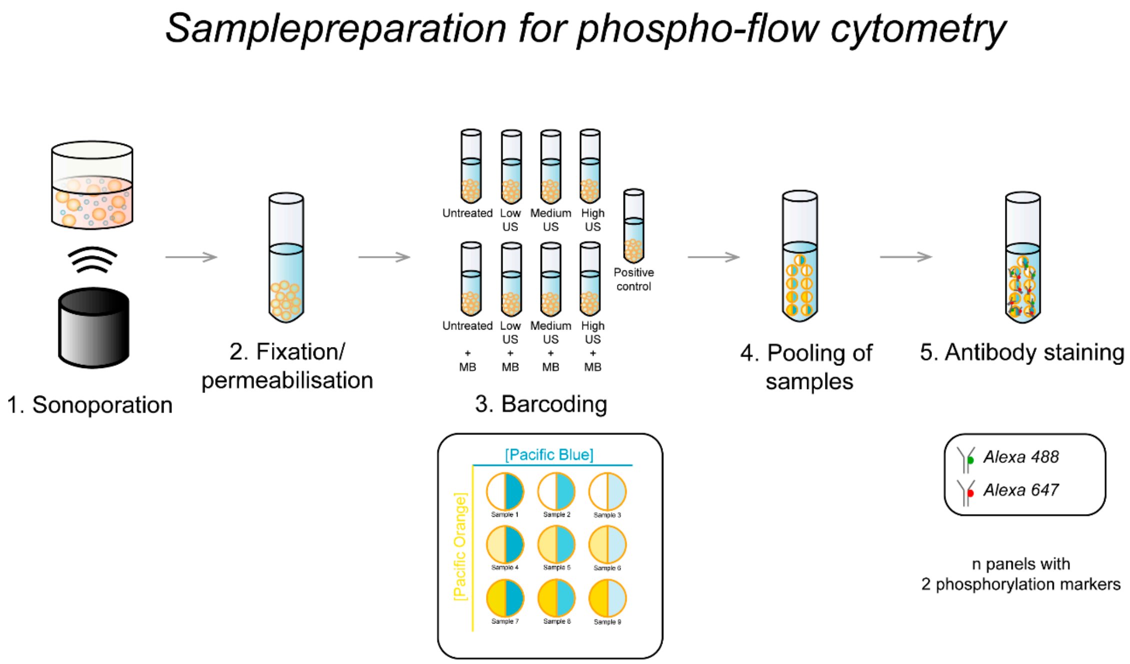

Figure S4. Sample processing for phospho-flow cytometry. After sonoporation cells were fixed and permeabilised, barcoded, stained with phosphorylation specific antibodies and analysed by flow cytometry. Barcoding of cells allows for reduced analytical variation. Samples harvested after 5 min, 30 min and 2 h after sonoporation at low medium and high ultrasound intensity were barcoded and pooled in to one sample per timepoint. All treated samples were normalized to untreated cells in the same barcode. Pooled samples were divided in tubes for antibody staining with panels consisting of 2 phosphospecific antibodies conjugated to respectively Alexa 488 and Alexa 647.

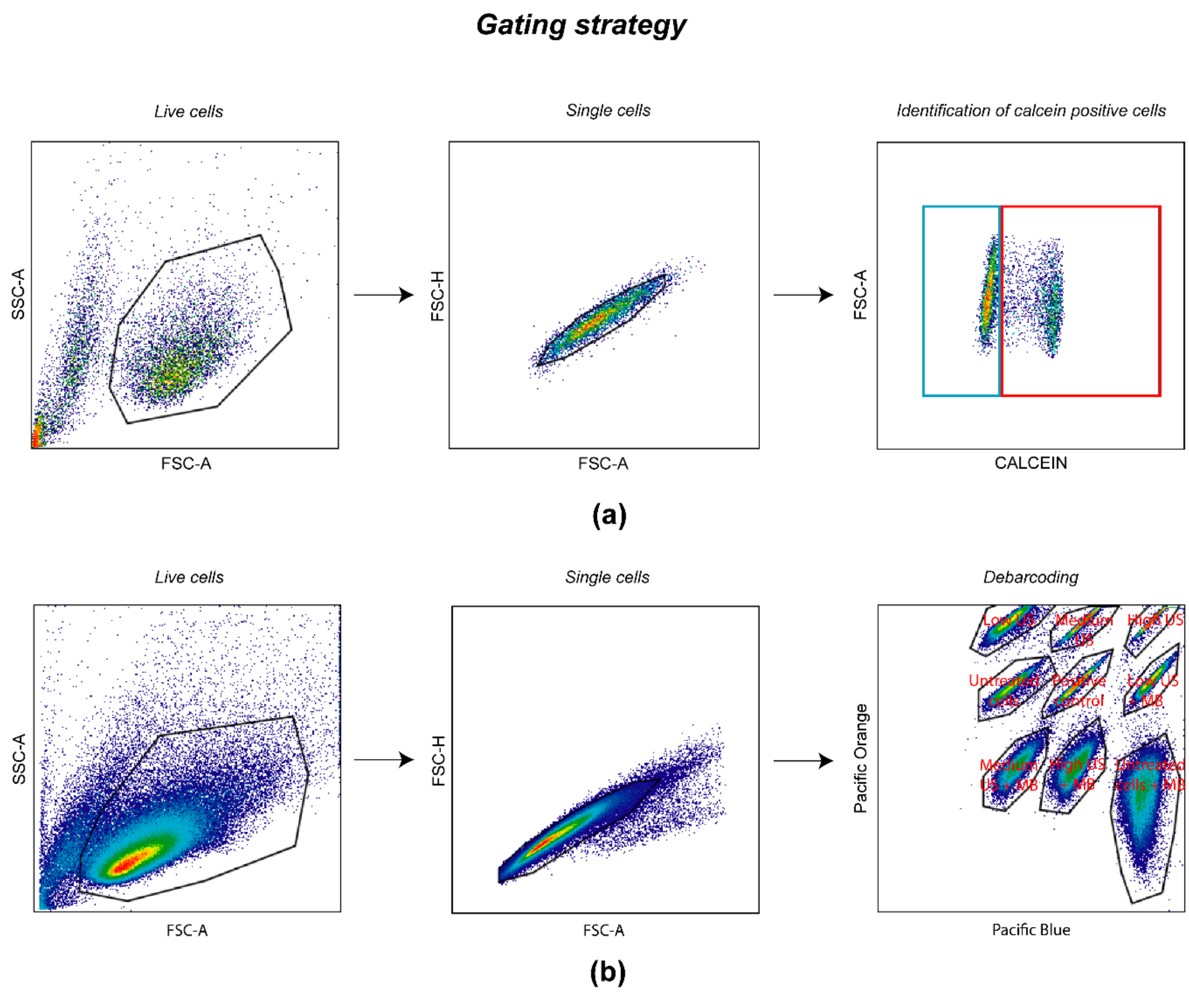

Figure S5. Gating strategy of flow cytometric data. After identification of the populations the median fluorescence intensity of each sample of cells treated with ultrasound ± microbubbles was compared to the median fluorescence intensity of the untreated cells. (**a**) Gating strategy for identification of calcein-positive population. One sample were analysed per treatment condition (**b**) Gating strategy for de-barcoding for identification of each individually stained sample. One pooled sample (untreated cells, low US, medium US, high US, positive control, low US + MB, medium US + MB, high US + MB) was analysed per timepoint post sonoporation.

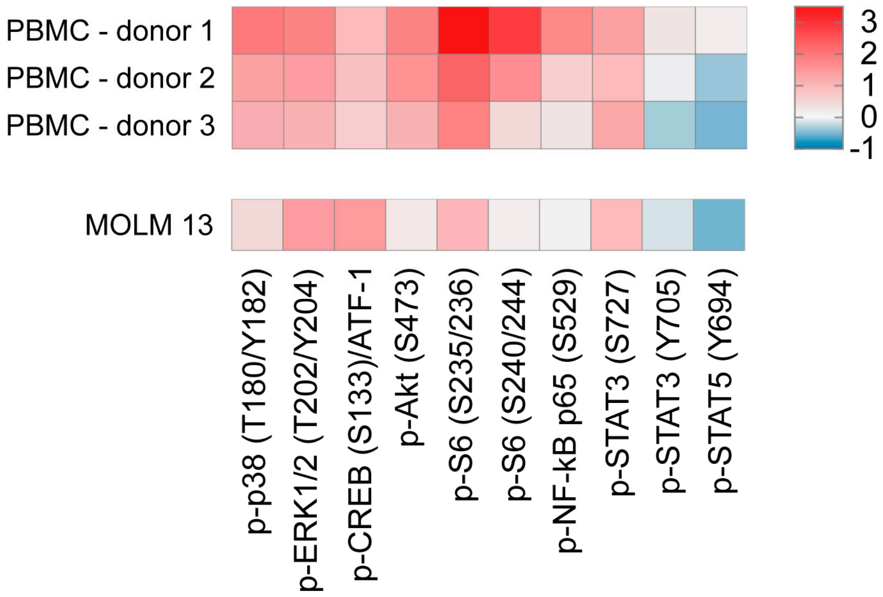

Figure S6. Change in phosphorylation status in response to positive controls (A23187 + PMA), represented as arcinhratio normalised to untreated cells. Some variation in response is observed between peripheral blood mononuclear cells (PBMC) from 3 different donors. PBMCs exhibit a stronger reponse to this stimuli compared to cell line MOLM-13.

Table S1. Phospho-proteins investigated using flow cytometry.

Target	Epitope	Fluorochrome	Manufacturer
Phospho-ERK1/2	Thr 202/Tyr204	Alexa 488	BD BioSciences
Phospho-p38	Thr 180/Tyr182	Alexa 488	BD BioSciences
Phospho-CREB	Ser 133/ATF-1	Alexa 488	BD BioSciences
Phospho-Akt	Ser 473	Alexa 647	BD BioSciences
Phospho-S6	Ser 235/236	Alexa 647	BD BioSciences
Phospho-S6	Ser 240/244	Alexa 488	BD BioSciences
Phospho-NF-kB p65	Ser 529	Alexa 647	BD BioSciences
Phospho-STAT3	Ser 727	Alexa 647	BD BioSciences
Phospho-STAT3	Tyr 705	Alexa 488	BD BioSciences
Phospho-STAT5	Tyr 694	Alexa 647	BD BioSciences
Phospho-FAK	Ser 910	Alexa 488	BD BioSciences
Phospho-Src	Tyr 418	Alexa 488	BD BioSciences
Phospho-PDPK1	Ser 241	Alexa 488	BD BioSciences
Ack-53	Ack 382	Alexa 647	BD BioSciences
Phospho-53	Ser 37	Alexa 647	BD BioSciences

Table S2. The effect of increasing ultrasound intensity on uptake of calcein and viability (Ultrasound treatment vs untreated sample).

		MOLM-13				PBMC			
		**No US**	**Low US**	**Medium US**	**High US**	**No US**	**Low US**	**Medium US**	**High US**
**Calcein positive cells**	No MB	---	ns	ns	>0.001	---	ns	ns	ns
	Sonazoid™	ns	ns	>0.0001	>0.0001	ns	ns	=0.05	>0.0001
	SonoVue®	ns	ns	>0.0001	>0.0001				
**Calcein MFI fold change**	No MB	---	ns	ns	>0.001	---	ns	ns	>0.05
	Sonazoid™	>0.001	>0.0001	>0.0001	>0.0001	ns	ns	> 0.01	>0.0001
	SonoVue®	ns	>0.05	>0.0001	>0.0001				
**Cell count**	No MB	---	ns	ns	ns	---	ns	ns	ns
	Sonazoid™	---	ns	>0.001	>0.01	---	>0.05*	>0.01*	ns
**Hoechst**	No MB	---	>0.01*	>0.01*	ns	---	ns	ns	ns
	Sonazoid™	---	ns	>0.001	>0.0001	---	ns	ns	ns
**Colony forming assay**	No MB	---	ns	ns	ns				
	Sonazoid™	---	>0.05	>0.05	>0.001				

* The differences in the measured values are very small and not considered relevant

Table S3. The effect of adding microbubbles on uptake of calcein and viability (Treatment without microbubbles vs treatment with microbubbles).

		**MOLM-13**		**PBMC**	
		**Sonazoid™**	**SonoVue®**	**Sonazoid™**	**SonoVue®**
**Calcein positive cells**	No US	ns	ns	ns	Not tested
	Low US	ns	ns	ns	Not tested
	Medium US	>0.0001	>0.0001	>0.01	Not tested
	High US	>0.0001	>0.0001	>0.0001	Not tested
**Calcein MFI fold change**	No US	>0.001*	ns	ns	Not tested
	Low US	>0.0001	ns	ns	Not tested
	Medium US	>0.0001	>0.0001	>0.05	Not tested
	High US	>0.0001	>0.0001	>0.0001	Not tested
		0 h	24 h	0 h	24 h
**Cell count**	No US	---	---	---	---
	Low US	ns	ns	ns	>0.05*
	Medium US	ns	>0.001	ns	>0.001*
	High US	ns	>0.001	ns	ns
**Hoechst**	No US		ns		ns
	Low US		ns		ns
	Medium US		>0.0001		ns
	High US		>0.0001		ns
		7–10 days			
**Colony forming assay**	No US	ns			
	Low US	ns			
	Medium US	>0.01			
	High US	>0.001			

* The differences in the measured values are very small and not considered relevant

Table S4. The effect of using different bubbles on calcein uptake (SonoVue® vs Sonazoid™).

		**MOLM-13**			
		No US	Low US	Medium US	High US
Calcein positive cells	MOLM-13	ns	ns	>0.0001	>0.05
Calcein MFI fold change	MOLM-13	ns	>0.05	>0.01	>0.0001

Table S5. The different response in different cell types on calcein uptake (MOLM-13 vs PBMCs).

		**No US**	**Low US**	**Medium US**	**High US**
Calcein positive cells	Sonazoid™	>0.0001	>0.0001	>0.001	>0.0001
Calcein MFI fold change	Sonazoid™	ns	>0.001	>0.0001	>0.0001

Table S6. The effect of increasing ultrasound intensity and microbubbles on protein phosphorylation (Ultrasound treatment vs untreated sample).

			MOLM-13				PBMC			
			**No US**	**Low US**	**Medium US**	**High US**	**No US**	**Low US**	**Medium US**	**High US**
	No MB	5 min	---	ns	ns	>0.01	---	ns	ns	ns
		30 min	---	ns	ns	ns	ns	ns	>0.05*	ns
p-p38		2 h	---	ns	ns	ns	ns	ns	>0.001*	ns
(T180/Y182)	Sonazoid™	5 min	ns	>0.01	>0.001	> 0.001	ns	ns	ns	ns
		30 min	ns	ns	ns	ns	ns	ns	ns	ns
		2 h	ns	ns	ns	ns	ns	ns	ns	ns
	No MB	5 min	---	ns	ns	ns	---	ns	ns	ns
		30 min	---	ns	ns	ns	ns	ns	ns	ns
p-ERK1/2		2 h	---	ns	ns	ns	ns	ns	>0.01*	ns
T202/Y204	Sonazoid™	5 min	>0.01*	ns	>0.00001	>0.0001	ns	ns	ns	ns
		30 min	ns	ns	>0.01*	>0.0001*	ns	ns	ns	ns
		2 h	ns	ns	ns	ns	ns	>0.01*	ns	ns
	No MB	5 min	---	ns	ns	ns	---	ns	ns	ns
		30 min	---	ns	ns	ns	ns	ns	ns	ns
p-CREB		2 h	---	ns	ns	ns	ns	>0.01*	ns	ns
S133/ATF-1	Sonazoid™	5 min	ns	ns	>0.001	>0.001	ns	ns	ns	ns
		30 min	ns	ns	>0.05	>0.05	ns	ns	ns	>0.05*
		2 h	ns	ns	>0.001	>0.05	>0.01*	ns	ns	ns

			MOLM-13				PBMC			
			No US	Low US	Medium US	High US	No US	Low US	Medium US	High US
p-Akt S473	No MB	5 min	---	ns	ns	ns	---	ns	ns	ns
		30 min	---	ns	ns	ns	ns	ns	ns	ns
		2 h	---	>0.05*	ns	ns	ns	ns	ns	ns
	Sonazoid™	5 min	ns	ns	> 0.01	>0.01	ns	ns	ns	ns
		30 min	>0.01*	ns	ns	>0.05*	ns	>0.05*	ns	ns
		2 h	ns	ns	ns	>0.01*	ns	ns	ns	ns
p-S6 S235/236	No MB	5 min	---	ns	ns	ns	---	ns	ns	ns
		30 min	---	ns	ns	ns	ns	ns	ns	ns
		2 h	---	ns	ns	ns	ns	ns	ns	ns
	Sonazoid™	5 min	ns	ns	ns	ns	ns	ns	ns	ns
		30 min	ns	ns	ns	ns	ns	>0.05*	>0.01*	ns
		2 h	ns	ns	ns	ns	ns	ns	ns	ns
p-S6 S240	No MB	5 min	---	ns	ns	ns	---	ns	ns	ns
		30 min	---	ns	ns	ns	ns	ns	ns	ns
		2 h	---	ns	ns	ns	ns	ns	>0.05*	ns
	Sonazoid™	5 min	ns	ns	ns	ns	ns	ns	>0.0001*	ns
		30 min	ns	ns	ns	ns	ns	ns	ns	ns
		2 h	ns	ns	ns	ns	ns	>0.001*	ns	ns
	No MB	5 min	---	ns	ns	ns	---	ns	ns	ns

			MOLM-13				PBMC			
			No US	Low US	Medium US	High US	No US	Low US	Medium US	High US
p-NF-kB p65 S529		30 min	---	ns	ns	ns	ns	ns	ns	ns
		2 h	---	ns	ns	ns	ns	ns	ns	ns
	Sonazoid™	5 min	ns	ns	ns	>0.01*	ns	ns	ns	ns
		30 min	ns		>0.01*	ns	ns	ns	ns	ns
		2 h	ns	ns	ns	ns	ns	ns	ns	ns
p-STAT3 S727	No MB	5 min	---	ns	ns	ns	---	ns	ns	ns
		30 min	---	ns	ns	ns	ns	ns	ns	ns
		2 h	---	ns	ns	ns	ns	ns	ns	ns
	Sonazoid™	5 min	ns	ns	>0.0001	>0.0001	ns	ns	ns	ns
		30 min	ns	ns	>0.05*	ns	ns	ns	ns	>0.01*
		2 h	ns	ns	>0.05*	ns	ns	ns	ns	ns
p-STAT3 Y705	No MB	5 min	---	>0.01*	>0.01*	ns	---	ns	ns	ns
		30 min	---	ns	ns	ns	ns	ns	ns	ns
		2 h	---	ns	ns	ns	ns	ns	ns	ns
	Sonazoid™	5 min		>0.01*	ns	ns	ns	ns	ns	ns
		30 min		>0.01*	ns	ns	>0.01*	ns	ns	ns
		2 h		ns	ns	ns	ns	ns	ns	ns
p-STAT5 Y694	No MB	5 min	---	**	**	**	---	ns	ns	ns
		30 min	---	**	**	**	ns	ns	ns	ns

		MOLM-13				PBMC			
		**No US**	**Low US**	**Medium US**	**High US**	**No US**	**Low US**	**Medium US**	**High US**
	2 h	---	**	**	**	ns	ns	ns	ns
Sonazoid™	5 min		**	**	**	ns	ns	ns	ns
	30 min		**	**	**	ns	ns	> 0.01*	ns
	2 h		**	**	**	ns	ns	ns	ns

* The difference in the actual measured values are very small and not considered relevant (Arcinhratio below 0.2).

** STAT5 was unchanged, and only analyzed once for MOLM-13. Statistical significance could not be tested.

Phosphorylation of FAK, Src, PDPK-1 and p53 was unchanged. These were analyzed once for MOLM-13, and not at all for PBMCs. Statistical significance could not be tested.

Table S7. The effect of increasing ultrasound intensity protein phosphorylation (Ultrasound treatment X vs Ultrasound treatment Y).

			MOLM-13	
			**Low US *vs* Medium US**	**Medium US *vs* High US**
p-p38 (T180/Y182)	No MB	5 min	ns	ns
		30 min	ns	ns
		2 h	ns	ns
	Sonazoid™	5 min	>0.01	ns
		30 min	ns	ns
		2 h	ns	ns
p-ERK1/2 T202/Y204	No MB	5 min	ns	ns
		30 min	ns	ns
		2 h	ns	ns
	Sonazoid™	5 min	>0.0001	ns
		30 min	ns	ns
		2 h	ns	ns
p-CREB S133/ATF-1	No MB	5 min	ns	ns
		30 min	ns	ns
		2 h	ns	ns
	Sonazoid™	5 min	>0.01	ns
		30 min	ns	ns
		2 h	ns	ns
p-Akt S473	No MB	5 min	ns	ns
		30 min	ns	ns
		2 h	ns	ns
	Sonazoid™	5 min	>0.05	ns
		30 min	ns	ns
		2 h	ns	ns
p-S6 S235/236	No MB	5 min	ns	ns
		30 min	ns	ns
		2 h	ns	ns
	Sonazoid™	5 min	ns	ns
		30 min	ns	ns
		2 h	ns	ns
p-STAT3 S727	No MB	5 min	ns	ns
		30 min	ns	ns
		2 h	ns	ns
	Sonazoid™	5 min	>0.001	ns
		30 min	ns	ns
		2 h	ns	ns
